# Anti-inflammatory effects and possible mechanism of action of lupeol acetate isolated from *Himatanthus drasticus *(Mart.) Plumel

**DOI:** 10.1186/1476-9255-7-60

**Published:** 2010-12-17

**Authors:** Daniel L Lucetti, Elaine CP Lucetti, Mary Anne M Bandeira, Helenicy NH Veras, Aline H Silva, Luzia Kalyne AM Leal, Amanda A Lopes, Victor CC Alves, Gabriela S Silva, Gerly Anne Brito, Glauce B Viana

**Affiliations:** 1 Department of Physiology and Pharmacology, Federal University of Ceará, Brazil; 2 Department of Pharmacy, Federal University of Ceará, Brazil; 3 Department of Morphology, Federal University of Ceará, Brazil; 4 Faculty of Medicine of Juazeiro do Norte, Ceará, Brazil

## Abstract

**Background:**

The species *Himatanthus drasticus *is popularly known in Northeast Brazil as "janaguba" and belongs to the family Apocynaceae. The latex collected from its stem bark is used for several purposes including anti-inflammatory properties and presents among its bioactive constituents the pentacyclic triterpene lupeol. The objective of the present work was to study *in vivo *and *in vitro *the lupeol acetate (LA) isolated from the plant latex, in several models of inflammation.

**Methods:**

Male Swiss mice (25-30 g, 6-24 animals per group) were administered with LA, 30 min before the test initiation. In the evaluation of analgesic activity the formalin test was used. The anti-inflammatory activity was evaluated by the following tests: paw edema induced by carrageenan and dextran, and the carrageenan-induced neutrophil migration into peritoneal cavities. Furthermore, the effect of LA on the myeloperoxidase release (MPO, an inflammation biomarker) from human neutrophils was also determined, as well as its antioxidant potential by the DPPH assay.

**Results:**

In the formalin test, LA (10, 25 and 50 mg/kg, i.p.) inhibited both the 1^st ^(neurogenic, 0-5 min) and mainly the 2^nd ^(inflammatory, 20-25 min) phase. Naloxone completely reversed the LA effect, indicating the participation of the opioid system. LA also significantly inhibited carrageenan- and dextran-induced paw edemas, as well as the neutrophil migration to the peritoneal cavity evaluated by the carrageenan-induced pleurisia. In this model, the effect of a very low dose of LA (0.1 mg/kg) was potentiated by the same dose of pentoxifylline (PTX), a known TNF-alpha inhibitor. LA (25 and 50 μg/ml) was also very effective in inhibiting MPO released from stimulated human neutrophils, and significantly decreased the number of cells expressing iNOS activity in the paw of mice submitted to carrageenan-induced edema, suggesting a drug involvement with the NO system.

**Conclusions:**

The anti-inflammatory effect of LA probably involves the opioid system, as indicated by the complete blockade of the opioid antagonist naloxone. Furthermore, the LA effect was potentiated by PTX (a TNF-alpha inhibitor). LA also decreased the number of iNOS cells, suggesting the participation of pro-inflammatory cytokines and the NO system in the drug action.

## Background

Through years of ingenious syntheses and structural modifications that usually follow the design and development of new drugs, many non-steroidal anti-inflammatory agents (NSAIDS) have been prepared and marketed [1]. However, these drugs are known to provoke adverse effects such as gastrointestinal irritations. Hence, the search for alternative anti-inflammatory drugs mainly from natural herbs is required.

The species *Himatanthus drasticus*, popularly known in Northeast Brazil as "janaguba", belongs to the family Apocynaceae. It is a tree that grows up to 7 m in height, with dense foliage at the ends of its branches. Its geographical distribution extends from Southeast Brazil to French Guyana, Suriname and Guyana. In Brazil, it occurs in several states, especially from the Northeastern region. The latex from its stem bark is over-exploited without control by local Brazilian communities, for instance in the Northeasterm region of Cariri for medicinal purposes, mainly for the treatment of tumours, inflammatory processes and ulcers [2].

The latex from several species of the *Himatanthus *genus including *H. drasticus *is rich in triterpenes. These are molecules formed by thirty carbon atoms and six isoprenoid units (with five carbon atoms each) [3]. The triterpenes are divided into several families with different base structures. Lupeol, betulin, betulinic acid and calenduladiol are triterpenes belonging to the lupane family. As far as their biological activities are concerned, the pentacyclic triterpenes including lupeol are a group of promising secondary plant metabolites [4].

Lupeol is an important constituent of the species *H. drasticus *and may be closely related to its anti-inflammatory action. Besides pentacyclic triterpenes, *H. sucuuba *is another species reported to present depsides, iridoides and alkaloids as well [5]. Furthermore, this species has been much more studied than *H. drasticus *what indicates the importance of knowing more and better about its bioactive constituents. Based on the popular use of *H. drasticus*, due to its antitumor, antifungal and anti-inflammatory actions [6], studies with this plant have been intensified. The *H. drasticus *latex protein has no cytotoxic effect *in vitro *or hemolytic character, but has antitumor effects *in vivo *[7].

The present research is aimed at evaluating the anti-inflammatory activities of lupeol acetate (LA) isolated for the first time from the latex of *H. drasticus*, on several models of experimentally induced inflammation in mice. Besides, the activity of LA on the MPO release from human neutrophils was also evaluated. MPO is released by activated neutrophils, and is a biomarker for inflammation. Furthermore, to clarify the LA mechanism of action, we studied the participation of pentoxifylline, a PDE5 and TNF-alpha inhibitor, and morphine, a mu and kappa agonist, on inflammatory processes, and their possible interaction with LA. Finally, histological studies (HE staining) and the effects of LA on TNF-alpha and iNOS were assessed by immunohistochemistry on the inflamed mouse paw, in the carrageenan-induced edema model.

## Materials and methods

### Preparation and chemical characterization of lupeol acetate (LA)

The *H. drasticus *latex was collected at the "Chapada do Araripe" region (South of Ceará state) by permission of the Brazilian Institute for the Environment and Renewable Natural Resources (IBAMA). The identification of the plant was carried out through *exsiccatae* which were subjected to comparison with the one already registered (n° 31685) at the Prisco Bezerra Herbarium of the Federal University of Ceará (UFC).

Initially, the latex was submitted to a five-time extraction with ethyl acetate. The ethyl acetate extract was evaporated at room temperature, and then subjected to a corn starch column chromatography under pressure. A dichloromethane/acetone mixture with increasing polarity was used as the eluent, after what a 10% yield (1 liter of latex = 10 g) whitish solid was obtained. Next, the solid was subjected to purification on a silica column, using as eluent a mixture of hexane/dichloromethane in increasing polarity. This silica purification process gave 120 fractions which were analyzed by thin-layer chromatography (eluent: dichloromethane; revelation: UV lamp and iodine). The final purification resulted in a white and crystalline solid compound with a 93.2% purity, as determined by gravimetric analyses. Its structure was established on the basis of spectroscopic data analysis and by comparison with the literature data. NMR ^13^C data from these crystals demonstrated that they are predominantly lupeol acetate when compared to the literature data [5]: (δ_C1 _38,6; δ_C2 _23,9; δ_C3 _81,2; δ_C4 _38,0; δ_C5 _55,5; δ_C6 _18,2; δ_C7 _34,4; δ_C8 _41,0; δ_C9 _50,5; δ_C10 _37,2; δ_C11 _21,1; δ_C12 _25,2; δ_C13 _38,2; δ_C14 _43,0; δ_C15 _27,6; δ_C16 _35,7; δ_C17 _43,0; δ_C18 _48,4; δ_C19 _48,2; δ_C20 _151,1; δ_C 21 _29,9; δ_C22 _40,2; δ_C23 _28,1; δ_C24 _16,7; δ_C25 _16,2; δ_C26 _16,8; δ_C27 _14,7; δ_C28 _18,2; δ_C29 _109,6; δ_C30 _19,4; δ_C1' _171,2; δ_C2' _21,1).

### Drugs

Carrageenan (lambda type IV), dextran sulfate, naloxone and indomethacin were purchased from Sigma Chemical (St. Louis, MO, USA). Dexamethasone was from Aché (Brazil), heparin from Wyeth (Brazil), morphine from Cristália (Brazil) and pentoxifylline from Sanofi-Aventis (Brazil). All other reagents were of analytical grade. The lupeol acetate (LA) was dissolved in an aqueous solution of 1% Tween 80 (Sigma, USA), and indomethacin was dissolved in carboxy-methylcellulose before use.

### Animals

Male Swiss mice (25-30 g) were provided by the Animal House of the Federal University of Ceará, Brazil. The animals were housed into plastic cages with sawdust as beddings, and kept in a room with controlled temperature (25 ± 2°C) under a 12/12 h light/dark cycle and food and water supplied *ad libitum*. The experiments were carried out according to the Guide for the Care and Use of Laboratory Animals of the U.S. Department of Health and Human Services (NIH publication no. 85-23, revised 1985). The project was previously approved by the Animal's Ethics Committee of the Faculty of Medicine of the Federal University of Ceará.

### Formalin test in mice

Twenty microliters of 1% formalin were administered (s.c.) in the mouse's right hind paw, and the licking time was recorded from 0 to 5 min (phase 1, neurogenic) and from 20 to 25 min (phase 2, inflammatory), after the formalin injection [8]. The animals were treated with saline (0.1 mL/10 g, i.p.), morphine (7.5 mg/kg, i.p.), LA (10, 25, and 50 mg/kg, i.p.), morphine + naloxone (7.5 and 2 mg/kg, i.p, respectively) or LA + naloxone (50 and 2 mg/kg, i.p., respectively), 30 min before the formalin injection.

### Carrageenan-induced mice paw edema

Carrageenan-induced paw inflammation was achieved according to the method described previously [9]. The animals were randomly selected and divided into groups of 6-23 animals. LA was dissolved in 1% Tween 80, and administered at the doses of 2, 10, 20 and 50 mg/kg, i.p. The other groups were injected with the reference drug (indomethacin, 10 mg/kg, i.p.) or vehicle (Tween 80). Thirty minutes later, the edema was induced by the injection of 50 μL of 1% v/v carrageenan solution into the animal's right hind paw. Measurements of the paw volume were done by means of a plethysmometer (Ugo Basile, Italy), immediately prior to the carrageenan injection and after 1, 2, 3, 4 and 24 h. The paw edema volume was determined by the difference between the final and initial volumes.

### Dextran-induced mice paw edema

The treatment of animals and measurements of the paw volume (0, 1, 2, 3 and 4 h) were done as described above. An injection of dextran (100 μg/0,1 ml/paw) was used [10]. LA (12.5 and 25 mg/kg, i.p.), dexamethasone (1.5 and 3 mg/kg, i.p.) and vehicle (1% Tween 80 solution) were administered to the different groups of mice, 30 min prior to the dextran injection.

### *In vivo *carrageenan-induced neutrophil migration into mice peritoneal cavities

Groups of 8 animals were treated with LA (0.1, 1, 10 and 20 mg/kg, i.p.), dexamethasone (5 mg/kg, i.p.) or vehicle, 30 min before the induction of inflammation by means of 1% carrageenan (500 μg/mL). The test was developed according to the experimental protocol described below [11]. All drugs were administered at a volume of 10 mL/kg, and then the animals were returned to their cages with free access to water. After five hours, the peritoneal fluid was collected by abdominal laparoscopy. For this, all animals were pretreated with a heparinized saline (5 IU/ml, ip). A sample of the peritoneal fluid was diluted 1:10 in Turk liquid for quantification of cell number, using a Neubauer chamber. For differential counting of leukocytes, the exsudate was centrifuged at 1,000 rpm for 5 min, and 200 μL of 3% bovine serum albumin were added to the pellet for the preparation of slides. The cells were stained by a conventional fast pigment, and the results expressed by the number of cells/mm^3 ^(total and differential leukocyte counts in the wash fluid).

### Myeloperoxidase (MPO) release from human neutrophils

According to previous methods [12], 2.5 × 10^6 ^cells were suspended in buffered Hank's balanced solution, containing calcium and magnesium. The preparations contained predominantly neutrophils (85.0 ± 2.8%), and the cell viability was 97.7 ± 0.94% as determined by the Trypan-blue test. The cells were incubated with LA (0.1, 1 and μg/mL) for 15 min at 37°C, and stimulated by the addition of phorbol myristate acetate (PMA, 0.1 μg/mL) for 15 min at 37 °C. The suspension was centrifuged for 10 min at 2,000 × g at 4°C. Aliquots (50 μL) of the supernatants were added to phosphate-buffered saline (100 μL), phosphate buffer (50 μL, pH 7.0) and H_2_O_2 _(0.012%). After 5 min at 37°C, thiamine monophosphate (TMP, 1.5 mM, 20 μL) was added, and the reaction stopped by 30 μL of a sodium acetate solution (1.5 M, pH 3.0). The absorbance was determined in triplicates using a spectrophotometer (620 nm).

### LDH release from human neutrophils

After isolation, a suspension of cells (5.0 × 10^6^/mL) was incubated with LA (1 to 50 μg/mL), vehicle or 0.2% Triton X-100 (known to cause cell lysis and used as a positive control), for 15 min at 37°C. Then, the LDH release was determined according to the manufacturer's instructions (LDH liquiform of Labtest Diagnosis, MG, Brazil). The increasing LDH leakage was expressed by the absorbance decrease at 340 nm.

### *In vitro *determination of the antioxidant activity by the DPPH (1, 1-diphenyl-2-picryl-hydrazyl) assay

The antioxidant activities of LA and alpha-tocopherol (as standard) were determined by the DPPH assay [13]. Briefly, 0.1 mL alpha-tocopherol (from a 3 mg/mL solution, final concentration of 50 μg/mL) or LA (1, 2.5, 5 and 10 μg/mL) were placed into test tubes, followed by the addition of 3.9 mL 0.3 mM DPPH (in a 1:1 methanol solution). LA, alpha-tocopherol or vehicle (30% DMSO in a methanol:ethanol 1:1 solution) were vigorously shaken with DPPH and left standing for 60 min in the dark. A 0.1 mL methanol:ethanol solution was used for blank. The reduction of DPPH was spectrophotometrically determined at 517 nm. The radical scavenging activity (RSA) was calculated as the percentage of the DPPH discoloration, by the equation: % RSA = [(A_DPPH - _A_s _)/A_DPPH_] × 100, where A_s _is the absorbance of the test solution, when the compound has been added, and A_DPPH _is the absorbance of the DPPH solution.

### Immunohistochemistry analyses for TNF-α and iNOS

For immunohistochemistry assays of the tumor necrosis factor-alpha (TNF-α) and induced nitric oxide synthase (iNOS), the streptavidine-biotin-peroxidase method was used [14]. Three groups of mice were treated with distilled water; two other groups were treated respectively with LA (50 mg/kg, i.p.) and indomethacin (10 mg/kg, i.p.). After 30 min, the animals were administered with an intraplantar injection of carrageenan. Three hours later, they were sacrificed and 5 mm plantar region sections of the carrageenan-injected hind paw were immersed in 10% formalin for 24 h and inserted in paraffin blocks. The sections were then deparafinized, dehydrated in xylol and ethanol, and immersed in 0.1 M citrate buffer (pH 6) under microwave heating for 18 min, for antigen recovery. After cooling at room temperature for 20 min, the sections were washed with a phosphate buffered saline (PBS) solution, followed by a 15 min blockade of endogenous peroxidase with a 3% H_2_O_2 _solution. The sections were incubated overnight (4°C) with rabbit primary antibodies (anti-TNF-α or anti-iNOS, respectively) as 1:200 or 1:400 dilutions in PBS-BSA. At the next day, the sections were washed in PBS and incubated for 30 min with the secondary biotinilated rabbit antibody (anti-IgG), 1:200 dilution in PBS-BSA. After washing in PBS, the sections were incubated for 30 min with the conjugated streptavidin peroxidase complex (ABC Vectastain^® ^complex, Vector Laboratories, Burlingame, CA, USA). After another washing with PBS, the sections were stained with 3,3'diaminobenzidine-peroxide (DAB) chromophore, counter-stained with Mayer hematoxylin, dehydrated and mounted in microscope slides for analyses.

### Statistical analysis

All results are presented as mean ± S.E.M. One-way ANOVA followed by the Student-Newman-Keuls test were used for comparing the results among treatments. The significance level was set at p < 0.05.

## Results

### Formalin test in mice

LA (10, 25 and 50 mg/kg, i.p.) reduced both phases of the formalin test, and the results were significant at the two higher doses. However, the effects were mainly on the 2^nd ^phase with 61% inhibition, whereas the 1^st ^phase was inhibited by 41% at the LA dose of 50 mg/kg, i.p. The naloxone pretreatment completely reversed the LA effect, in the 1^st ^and 2^nd ^phases, indicating the participation of the opioid system in LA antinociceptive and anti-inflammatory actions. As expected, morphine used as the reference drug significantly decreased the 1^st ^(63%) and 2^nd ^(91%) phases of the test, and had its effect on both phases also reversed by naloxone. The data are presented in Table 1.

**Table 1:** The effect of lupeol acetate (LA) on pain behavior in the formalin test

Group	Paw licking (s)			
	1^st ^phase	Inhibition	2^nd ^phase	Inhibition
**Control**	64.0 ± 2.9	-	32.6 ± 3.9	-
**Morphine**				
(7.5 mg/kg, i.p.)	23.8 ± 2.8^a^	62.8	2.9 ± 1.6^a^	91.1
**LA **(mg/kg, i.p.)				
10	55.8 ± 3.6	12.8	41.2 ± 5.3	-
25	50.3 ± 4.7^a ^	21.4	13.8 ± 3.9^a^	57.6
50	36.1 ± 2.2^a^	46.5	12.6 ± 2,2^a ^	61.3
**Morphine + Naloxone**				
(7.5 + 2 mg/kg, i.p.) LA + Naloxone	51.5 ± 5.3^b ^	19.5	29.1 ± 2.4^b^	10.7
(50 + 2 mg/kg, i.p.)	56.4 ± 2.0^c ^	11.9	29.1 ± 2.5^c ^	10.7

Pain response was recorded between 0-5 (1^st ^phase) and 20-25 min (2^nd ^phase). LA was administered 30 min before formalin. **a**. vs. control; **b**. vs. morphine; **c**. vs. LA 50, at p < 0.05 (ANOVA followed by Student-Newman-Keuls as the *post hoc *test).

### Carrageenan-induced mouse paw edema

**Figure 1:**
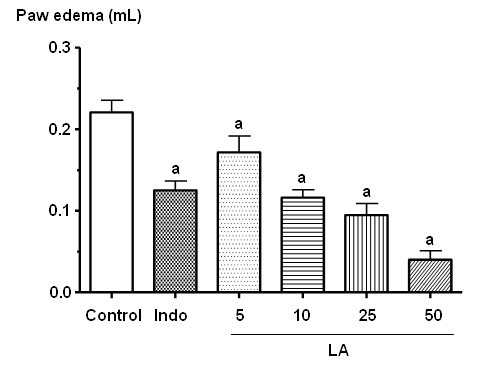
**Effects of lupeol acetate (LA: 5, 10, 25 and 50 mg/kg, i.p.) or indomethacin (10 mg/kg, i.p.) on the paw edema induced by carrageenan, at the 3^rd ^h**. Each value represents the mean ± S.E.M. of 7-23 animals per group. **a**. vs. control, at p < 0.05 (ANOVA followed by the Student-Newman-Keuls as the *post hoc *test).

The pre-treatment of mice with LA (2, 5, 10 and 20 mg/kg, i.p.) significantly reduced the volume (μL) of the edema, in the 1^st^, 2^nd^, 3^rd ^and 4^th ^hours after administration of carrageenan, as compared to the control group. The doses that showed greater effects were 10 and 20 mg/kg, which reduced the edema by 40 (1^st ^h), 39 (2^nd ^h), 45 (3^rd ^h), 51% (4^th ^h) and 47 (1^st ^h), 47 (2^nd ^h), 43 (3^rd ^h), 49% (4^th ^h), respectively. Figure 1 shows the LA effect at the 3^rd ^h, corresponding to its maximum activity.

### Dextran-induced mouse paw edema

**Figure 2:**
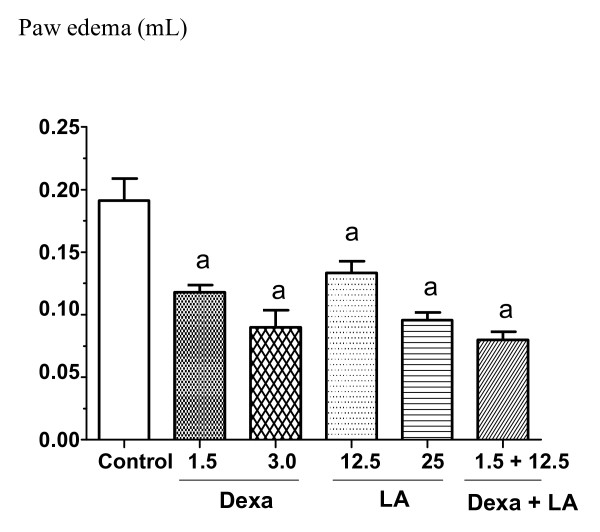
**Effects of lupeol acetate (LA: 12.5 or 25 mg/kg, i.p.) or dexametasone (1.5 or 3 mg/kg, i.p.) on the paw edema induced by dextran, at the 3^rd ^h**. Each value represents the mean ± S.E.M. of 5-7 animals per group. **a**. vs. control, at p < 0.05 (ANOVA followed by the Student-Newman-Keuls as the *post hoc *test).

The pre-treatment of mice with LA (12.5 and 25 mg/kg, i.p) significantly reduced the volume (μL) of the edema in the 2^nd ^(31 and 41%), 3^rd ^(30 and 50%) and 4^th ^(23 and 27%) hours after administration of dextran, respectively, as compared to the control group. A group that had been treated with dexametasone (1.5 mg/kg, i.p.) was co-administered with LA at the dose of 12.5 mg/kg, i.p. This group had the volume (μL) of edema, on the 2^nd^, 3^rd ^and 4^th ^hours after the administration of dextran, reduced in 49, 58 and 52%, respectively. Figure 2 shows LA effects at the 3^rd ^h of development of the dextran-induced paw edema.

### Peritonitis induced by carrageenan in mice

**Figure 3:**
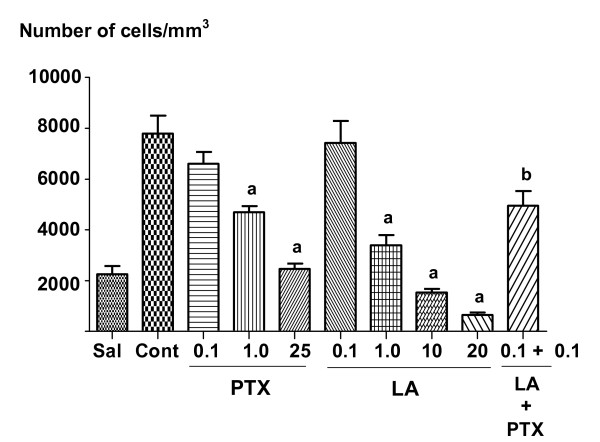
**Effects of the administration of lupeol acetate (LA: 0.1, 1, 10 and 20 mg/kg, i.p.) or pentoxifylline (0.1, 1 and 25 mg/kg, i.p.) on acute carrageenan-induced peritonitis, measured by the number of cells in the peritoneal fluid**. Each value represents the mean ± S.E.M. of 8 animals per group. **a**. vs. control, at p < 0.05 (ANOVA followed by the Student-Newman-Keuls as the *post hoc *test).

Figure 3 presents the LA effect on the carrageenan-induced pleurisia. Carrageenan (1%) caused a significant neutrophil migration when injected into the peritoneal cavity of mice. LA injected 30 min before carrageenan significantly decreased the carrageenan-induced neutrophil migration in a dose-dependent manner. The LA inhibitory effect against carrageenan-induced migration was about 52, 79 and 90%, at the doses of 1, 10 and 20 mg/kg, i.p., respectively. The reference drugs dexamethasone (5 mg/kg, i.p.) and pentoxifylline (1 and 25 mg/kg, i.p.) decreased the carrageenan-induced neutrophil migration by 82, 34 and 65%, respectively. The groups treated with a 0.1 mg/kg dose of pentoxifylline or LA showed no significant inhibition of neutrophils migration (15 and 5%, respectively), when compared to controls (in the presence of carrageenan only). However, when these drugs were co-administered at this same dose, they promoted a significant inhibition of 37%.

### LA effects on the myeloperoxidase (MPO) release from human neutrophils *in vitro*

**Figure 4:**
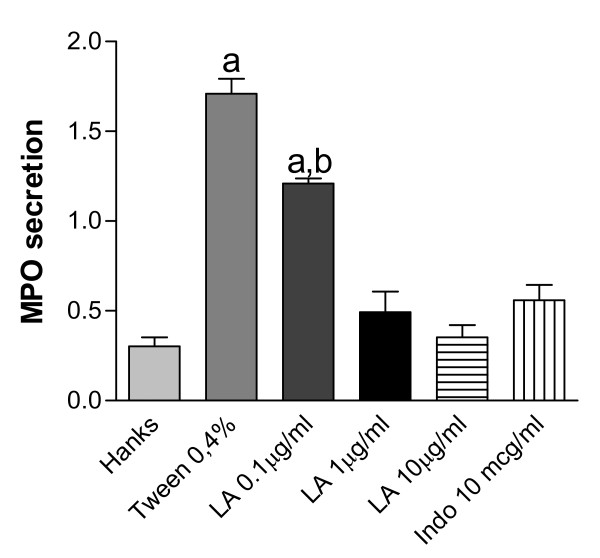
**Effects of lupeol acetate (LA: 0.1, 1 and 10 μg/ml) on PMA stimulated myeloperoxidase (MPO) activity from human neutrophils *in vitro***. The values are expressed as mean ± S.E.M. The analysis was done at least in quadruplicates and repeated in three different days. **a**. and **b**. vs. control (Hanks' solution) and vehicle (0.4% Tween 80), respectively, at p < 0.001 (ANOVA followed by the Student-Newman-Keuls as the *post hoc *test).

In order to evaluate the possible effect of LA on MPO, we determined its effects on the PMA-stimulated MPO release from human neutrophils. Our results showed (Figure 4) that a 5.7 times increase in enzyme release was observed in the presence of 0.4% Tween 80 (vehicle) as compared to Hanks' solution (negative control). On the other hand, significant and dose-dependent inhibitions were demonstrated with LA concentrations of 0.1, 1 and 10 μg/mL, and a maximal effect was already seen within this dose range. The effects observed with the two higher LA doses were similar to that of indomethacin (10 μg/mL) used as a reference drug.

### LA effects on the lactate dehydrogenase (LDH) release from human neutrophils *in vitro*

**Figure 5:**
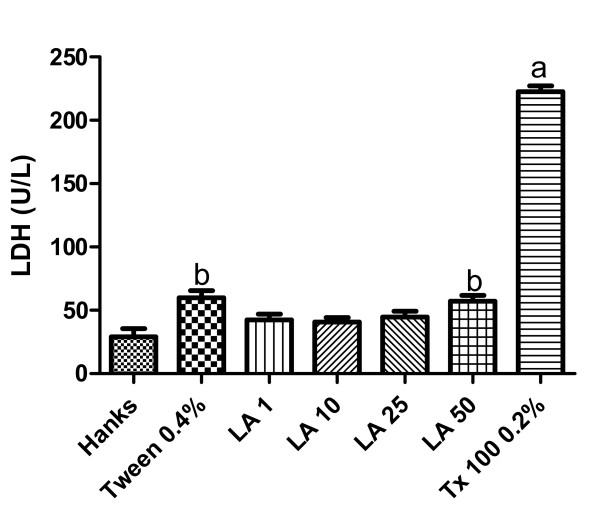
**Effects of lupeol acetate (LA: 10, 25, 50 μg/ml) on lactate dehydrogenase (LDH) release from human neutrophils *in vitro***. The values are expressed as mean ± S.E.M. The analysis was done at least in quadruplicates and repeated in three different days. **a**. and **b**. vs. control (Hanks' solution) and vehicle (0.4% Tween 80), respectively, at p < 0.01 to 0.001 (ANOVA followed by the Student-Newman-Keuls as the *post hoc *test).

The results of Figure 5 show that while Triton X-100 (a cytotoxic drug used as positive control) increases in 7.7 times LDH release from human neutrophils, the increase was only around 2 times in the presence of the vehicle (0.2% Tween 80) as related to Hanks' solution (negative control). On the other hand, while no significant enzyme release was observed with LA at the concentrations of 1, 10 and 25 μg/mL, a small but significant LDH release (around 2 times) was detected with the higher LA concentration (50 μg/mL), probably related to the presence of 0.2% Tween 80.

### LA shows no radical scavenging activity as evaluated by the DPPH *in vitro*

**Figure 6:**
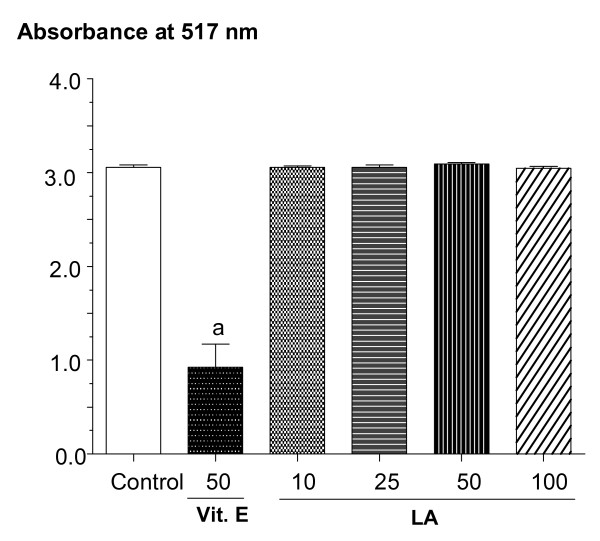
**DPPH radical scavenging activity of lupeol acetate (LA: 10, 25, 50 and 100 μg/ml) was measured at 517 nm, as compared to standard alpha-tocopherol (50 μg/ml)**. Values are means ± S.E.M. of triplicate experiments. **a**. vs. control, at p < 0.05 (ANOVA followed by the Student-Newman-Keuls as the *post hoc *test).

In order to detect any possible antioxidant effect of LA, the DPPH assay was performed. The results show that LA at the concentrations of 50, 100 and 200 μg/mL presents no radical scavenging capacity. On the contrary, vitamin E used as the reference drug significantly decreased the absorbance value, as related to controls (Figure 6).

### Histological analyses of mice paw in the carrageenan-induced edema model

**Figure 7:**
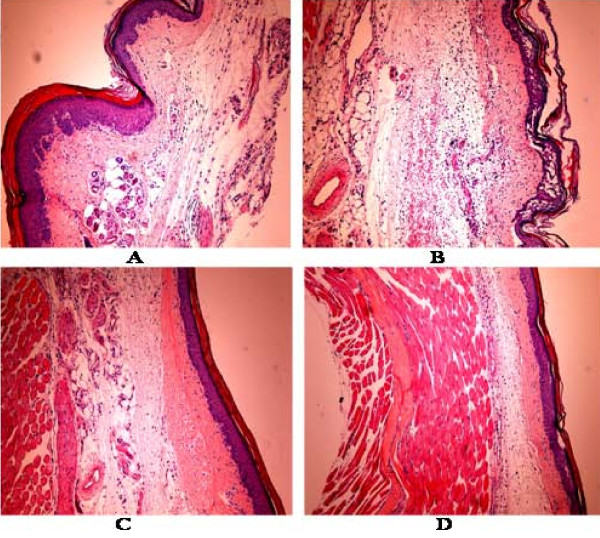
**Hematoxylin-eosin stained sections of paws from mice treated with lupeol acetate (LA), in the model of carrageenan-induced edema**. **A**: Control (0.04% Tween 80); **B**: Control + Carragenan; **C**: Indomethacin (10 mg/kg, i.p.) + Carrageenan; **D**: LA (50 mg/kg, i.p.) + Carrageenan. All figures were magnified by 100×.

The intraplantar injection of 1% carrageenan into the rat right hind paw produced an intense edema, characterized by epithelial and conjunctive tissue blisters and infiltrates of inflammatory PMN cells, mainly neutrophils, as compared to the carrageenan untreated group (normal control) (Figure 7). In the carrageenan groups pretreated with LA (50 mg/kg, i.p.) or indomethacin (10 mg/kg, i.p.) there were significant edema decreases as well as decreases in inflammatory cells infiltration.

### TNF-alpha immunohistochemistry and LA effects on mice paw in the carrageenan-induced edema model

**Figure 8:**
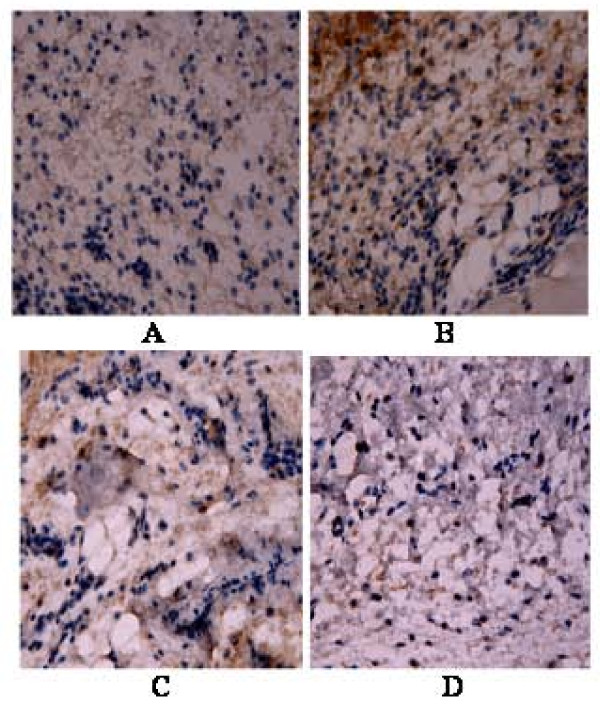
**Immunohistochemical staining for TNF-α of paws from mice treated with lupeol acetate (LA), in the model of carrageenan-induced edema**. **A**: Control (0.04% Tween 80); **B**: Control + Carrageenan; **C**: Indomethacin (10 mg/kg, i.p.) + Carrageenan; **D**: LA (50 mg/kg, i.p.) + Carrageenan. All figures were magnified by 400×.

Immunohistochemistry analyses showed a great number of cells expressing TNF-α in the paw conjunctive tissue, mainly mononucleated cells in mice submitted to carrageenan-induced inflammation, as compared to the untreated (normal controls) group (Figure 8). In the groups injected with carrageenan and pretreated with LA (50 mg/kg, i.p.) or indomethacin (10 mg/kg, i.p.), the reduction of TNF-α expressing cells was not significant.

### iNOS immunohistochemistry and LA effects on mice paw in the carrageenan-induced edema model

**Figure 9:**
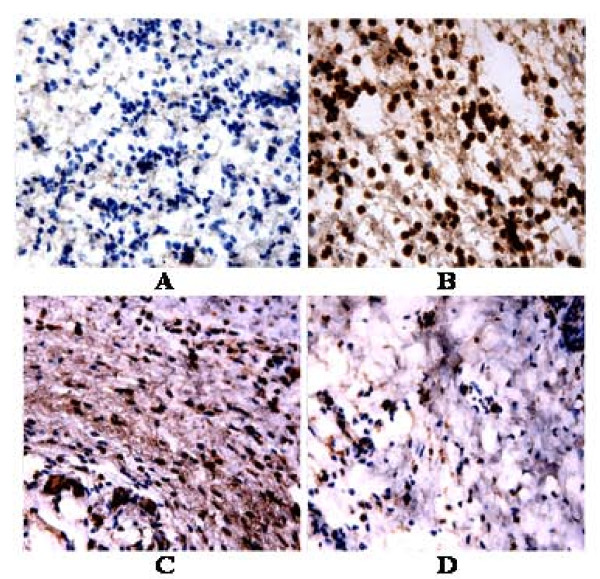
**Immunohistochemical staining for iNOS of paws from mice treated with lupeol acetate (LA), in the model of carrageenan-induced edema**. **A**: Control (0.04% Tween 80); **B**: Control + Carrageenan; **C**: Indometacin (10 mg/kg, i.p.) + Carrageenan; **D**: LA (50 mg/kg, i.p.) + Carrageenan. All figures were magnified by 400×.

A great number of iNOS expressing cells, mainly neutrophils in the conjunctive tissue, was observed in the inflamed paw after carrageenan administration, as related to the paw of untreated mice (normal controls) (Figure 9). In the carrageenan-treated groups pretreated with LA (50 mg/kg, i.p.) or indomethacin (10 mg/kg, i.p.), there were significant reductions of iNOS expressing cells.

## Discussion

Although the *Himatanthus *genus presents 14 species, distributed in tropical and sub-tropical areas, only 5 species were studied from chemical and/or biological points of view. In Brazil, these studies were carried out with species such as *H. sucuuba*, common to the Amazonian region. Furthermore, very few works are found in the literature on *H. drasticus*. The latex of these species is rich in triterpenes, including lupeol of a lupane type which was reported to present antitumor [15-18] and anti-inflammatory activities [19-22]. Also, a recent study [23] showed that the latex from *H. sucuuba *exhibited a potent leishmanicidal activity against intracellular amastigotes of *Leishmania amazonensis*, a causal agent of cutaneous leshmaniasis. Moreover, this latex also increased NO and TNF-alpha and decreased transforming growth factor-beta (TGF-beta) production in macrophages.

Lupeol is found in several other species and its antinociceptive and anti-inflammatory activities have been already demonstrated [24-28]. It is accepted that the anti-inflammatory property of lupeol often accompany its immune modulatory and anti-tumor action [29,30,4,15]. Despite the wealth literature data on lupeol, there are very few reports on lupeol acetate. It has been recently shown that lupeol acetate presents an anti-inflammatory activity by regulating TNF-alpha and IL-2 specific mRNA, besides upregulating the synthesis of IL-10 mRNA [31].

The latex from *H. drasticus *is widely used by communities from the Brazilian Northeastern region in gastritis and cancer among other health problems. In the present work, we showed that lupeol acetate (LA, 93.2% purity) isolated from the *H. drasticus *latex presented a potent anti-inflammatory action, in several models of inflammation in mice. Thus, LA inhibited predominantly the formalin test 2^nd ^phase, indicative of an inflammatory process. Interestingly, the LA effect was almost completely reversed by naloxone, suggesting that the effect is at least in part dependent upon the opioid system. The opioid participation in the LA action was further confirmed by the hot plate test, where its antinociceptive effect was as in the case of morphine also reversed by naloxone (data not shown).

LA significantly inhibited mice carrageenan- and dextran-induced paw edemas. However, it was more effective in the carrageenan model which induces paw edema and substantial leukocyte migration, mediated by histamine and serotonin in the initial phase of the inflammatory process, and by prostaglandin and bradykinin in later stages. On the other hand, paw edema induced by dextran although also mediated by histamine and serotonin does not involve leukocyte migration [9,32].

Lupeol administered topically has been shown [22] to suppress the mouse ear edema induced by 12-O-tetradecanoyl-phorbol acetate. Besides, lupeol significantly reduced PGE_2 _production from stimulated macrophages, *in vitro*. These authors concluded that lupeol possessed an anti-inflammatory activity which is probably related to its ability to prevent the production of pro-inflammatory mediators, such as TNF-α and IL-1β.

Furthermore, from a dose as low as 1 mg/kg, LA drastically and dose-dependently inhibited the neutrophils migration, as evaluated in the carrageenan-induced peritonitis model, corroborating its effect on the carrageenan-induced mice paw edema. Interestingly, in our work, LA effects were potentiated by PTX, a known TNF-alpha inhibitor [33]. We also showed that, in the mice paw submitted to carrageenan-induced edema, LA significantly decreased the edema and neutrophils migration, as compared to controls. This effect was similar to that of indomethacin, the reference drug, as assessed by histological techniques.

It has been observed that kappa-opioid drugs exert a powerful anti-inflammatory effect, reducing TNF-alpha release and expression, among other actions [34]. In addition, the expression of opioid receptors has been shown to occur during peripheral inflammation [35]. Considering that the LA effect was potentiated by PTX (an anti-TNF-α drug) and completely reversed by naloxone (an opioid antagonist), we could assume that at least in part LA acts inhibiting endogenous TNF-α. This cytokine is considered as a key factor in several inflammatory diseases and its regulation is mediated by transcription factors as the NF-kappaB. Previous studies [36] demonstrated glial activation and increased pro-inflammatory cytokines, in animal models of neuropathic pain. These authors showed that chronic propentofylline, a glial modulating and anti-inflammatory agent chemically similar to PTX, attenuated the development of hyperalgesia and restored the analgesic activity of acute morphine in neuropathic rats.

In an earlier study [37], the interactions among cytokines, PGE_2 _and cell migration during the various phases of carrageenan-induced acute inflammation were evaluated in the mouse air pouch model. These authors concluded that TNF-α seems to play an important role in this model, particularly for leukocyte migration in the 1^st ^phase of the inflammatory process. It was also demonstrated that PTX reduced histological lung injury and pulmonary neutrophil activity, in a model of hemorrhagic shock in rats [38], and the administration of PTX was associated with diminished NF-kappaB and enhanced CREB activation. In addition, in a model of experimental acute pancreatitis in rats [39], PTX significantly attenuated histological lung injury, pulmonary neutrophil activity and pro-inflammatory signaling.

We showed significant inhibitions of MPO release from human stimulated neutrophils by LA, at low concentrations (1 and 10 μg/mL) and effects were similar to those seen with indomethacin, used as reference drug. MPO is an enzyme stored in azurophilic granula of neutrophils, released after their activation and characterized by powerful pro-oxidative and pro-inflammatory proteins [40]. It is often used as a reliable biomarker of inflammation [41]. Recently [42], MPO was shown to promote lung neutrophilia and to influence indirectly subsequent chemokine and cytokine productions by other cell types in the lung. Furthermore, LA showed no significant cytotoxicity up to 50 μg/mL, as assessed by the LDH release from human neutrophils.

The administration of lupeol was reported to cause reductions of cellularity and eosinophils in the bronchoalveolar fluid, as assessed by a murine model of airway inflammation [21]. These authors showed that the treatment with lupeol reduced levels of IL-4, IL-5 and IL-13, characteristic of an allergic airway inflammatory process. Lupeol seems to be a potent anti-inflammatory and multi-target drug, targeting key molecular pathways such as those involving NF-kappaB, among others [43]. Previously [19], the lupeol treatment to mouse skin was reported to result in the inhibition of TPA-induced activation of several inflammatory and tumor-promoting factors, including NF-kappaB.

All together, our results showed that LA probably acts as an anti-inflammatory drug by decreasing the number of cells expressing iNOS. Although LA did not significantly decrease the number of cells expressing TNF-α, this effect becomes significant when LA is co-administered with PTX, a known TNF-α inhibitor. Other triterpenes were also shown to inhibit nitric oxide production by reducing iNOS expression [44], while a recent work [45] reported that the anti-inflammatory activity of these compounds is associated to the decreased production of iNOS and pro-inflammatory cytokines.

The oral administration of lupeol (25 to 200 mg/kg) was also reported to produce a dose-related inhibition of IL-2, IFN-γ and TNF-α, in mice pleural exudates [46]. Interestingly, PTX was shown to decrease lung MPO activity and NF-kappaB activation, in the model of LPS-induced acute lung injury in rats [47]. Finally, in the present work we showed that the anti-inflammatory effect of LA probably involves the opioid system and is potentiated by PTX. Furthermore, LA decreased the number of iNOS cells, suggesting that pro-inflammatory cytokines and the NO system play an active role in the drug action.

## Competing interests

The authors declare that they have no competing interests.

## Authors' contributions

DL and EL: carried out most of the *in vivo *experiments. MB, HV and AS: isolation and determination of the chemical structure of LA. LL, AL, VA and GS: responsible for all *in vitro *assays. GB: carried out all the immunohistochemistry assays. GV: participated in the design and coordination of the study. All authors read and approved the manuscript.
